# Limb-girdle muscular dystrophy type 2B misdiagnosed as polymyositis at the early stage: Case report and literature review

**DOI:** 10.1097/MD.0000000000010539

**Published:** 2018-05-25

**Authors:** Chuan Xu, Jiajun Chen, Yingyu Zhang, Jia Li

**Affiliations:** Department of Neurology (III), China-Japan Union Hospital of Jilin University, Changchun, Jilin Province, China.

**Keywords:** frame-shift mutation, immunohistochemistry, limb-girdle muscular dystrophy type 2B, polymyositis, Western-blot analysis

## Abstract

**Rationale:**

Dysferlin myopathy is an autosomal recessive hereditary muscular dystrophy due to deficiency of dysferlin caused by alteration of the DYSF gene; Limb-girdle muscular dystrophy type 2B (LGMD2B) is the most common in Its clinical phenotypes. However, LGMD2B is rarely seen in clinical cases and may initially present as weakness of proximalpelvis muscles and muscles in the posterior compartments of thighs,which will then cause difficulty in running and limping during walking. Laboratory tests at an early stage of the disease often indicate an increased level of serum creatine kinase (CK). Moreover, polymyositis (PM) is manifested as symmetrical proximal muscle weakness of the four limbs, accompanied by an increased level of serum CK. Thus, both are very difficult to identify in clinical practice.

**Patient concerns:**

A 25-year-old woman was admitted to our department as the limb weakness progressively worsened. She began to experience proximal muscle weakness of both lower limbs without obvious inducement, which markedly increased when she climbed the stairs or stood up after squatting. Then her symptoms worsened, with difficulty in proximal and distal lifting of the lower extremities.

**Diagnoses:**

Through combined immunohistochemistry and Western-blot analysis, The patient was diagnosed with LGMD2B.

**Interventions:**

There were symptomatic treatments such as coenzyme Q10.

**Outcomes:**

After symptomatic treatments, the patient's symptoms were obviously relieved, and the CK level decreased.

**Lessons:**

Through this case, we found that combined application of immunohistochemistry and Western-blot analysis is helpful in early diagnosis of LGMD2B, and a new site of frame-shift mutation in the patient's DYSF gene was found.

## 1 Introduction

Limb-girdle muscular dystrophy type 2B (LGMD2B) is a common clinical phenotype in dysferlinopathy. Its clinical features include weakness of proximal pelvis muscles and muscles in the posterior compartments of thighs, causing difficulty in standing after bending the knees. With disease progression, scapular band muscles and upper extremity muscles may also be involved, but with milder symptoms; facial, cervical, and hand muscles are generally unaffected.^[1]^ LGMD2B is an autosomal recessive disease caused by mutation of the *DYSF* gene.^[2]^*DYSF* gene encodes the dysferlin. Not only does dysferlin widely exist in cell membranes of skeletal and cardiac muscles, it also exists in the membranes of nonmyofiber cells, such as monocytes. In case of dysferlin deficiency, patients may have various symptoms, such as weakness of limbs.^[2]^ Polymyositis (PM) is an autoimmune disease mediated by cellular immunity. Common clinical symptoms include muscle weakness, elevation of muscle enzyme, etc. LGMD2B and PM are very similar in both clinical symptoms and histological staining of muscles, but differ in their management; hence, clinicians should be vigilant to avoid misdiagnosis. This study reports a case of LGMD2B misdiagnosed as PM at an early stage, and the inspirations from immunohistochemistry (IHC) and Western-blot (WB) analysis in early diagnosis and treatment of LGMD2B were summarized. The case report was approved by the Ethics Committee of China–Japan Union Hospital of Jilin University, and all examinations of the patient were approved by the patient herself. She also provided informed consent for publication of the findings.

## 2 Clinical data

The patient was a 25-year-old woman. Seven years ago, she began to experience proximal muscle weakness of both lower limbs without obvious inducement, which markedly increased when she climbed the stairs or stood up after squatting; there was no muscle weakness in the upper limbs, dysphagia, or other clinical manifestations. Her symptoms worsened, with difficulty in proximal and distal lifting of the lower extremities. She underwent multiple examinations for muscle enzyme levels, electromyography (EMG), etc., in another hospital and was diagnosed with “polymyositis.” She was treated with intermittent glucocorticoids and immunosuppressants. However, her symptoms gradually worsened. The patient denied family history of related genetic diseases.

The patient was admitted to our department 2 years ago as the above symptoms progressively worsened.

### 2.1 Neurological findings at admission

Muscle strength, muscular tension, and muscular volume of the upper limbs were normal. Muscle strength of the lower limbs was at level 4. Muscular tension in both lower limbs was normal. Distal muscular volume of the lower limbs was decreased with presence of muscular atrophy, which was more obvious on the left side. The patient was unable to perform coordinated movements during the physical examination. Reflexes of bilateral musculus biceps brachii and musculus triceps brachii were normal. Bilateral tendon reflex was lost. Bilateral Babinski signs were negative.

### 2.2 Findings during prior admissions

Creatine kinase level, 8448.2 U/L (26–140I U/L); erythrocyte sedimentation rate, 3 mm/h (0–20 mm/h); and C-reactive protein level, 0.170 mg/dL (0–0.8 mg/dL).

### 2.3 EMG findings 1 month prior to admission

When the left quadriceps femoris had a slight contraction, the duration of motor unit potential shortened, wave amplitude decreased, and the percentage of polyphasic wave increased, based on which the diagnostic indication was muscle-derived injury. In order to further clarify the diagnosis, electron microscopy of her left quadriceps femoris was conducted at our hospital, which indicated the following: main changes in the ultra-microstructure examination on skeletal muscles included muscular fiber atrophy, focal damage of the myofibril, obvious loose arrangement, presence of a small number of glycogen granules and fat droplets, a slightly increased number of mitochondria, and nuclear ingression, showing characteristics of pathologic changes of muscle diseases. There were fat droplets, increased number of mitochondria, and mitochondrial vacuolation in muscle fibers, indicating metabolic disorder of mitochondria and fat cells. However, no typical characteristic of pathological change in metabolic muscular disease was detected. Thickened basement membrane of blood vessels indicated possible immune mediation (skeletal muscle).

### 2.4 Immunohistochemical findings of the left quadriceps femoris muscle

**Figure 1:**
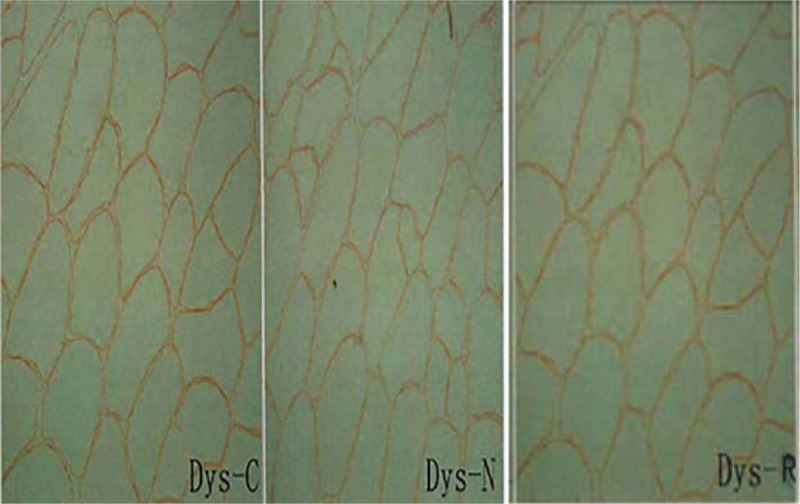
Dystrophin staining of muscle fiber in the left quadriceps of the patient indicated clear and continuous staining of Dys-C, Dys-N, and Dys-R in sarcolemma.

**Figure 2:**
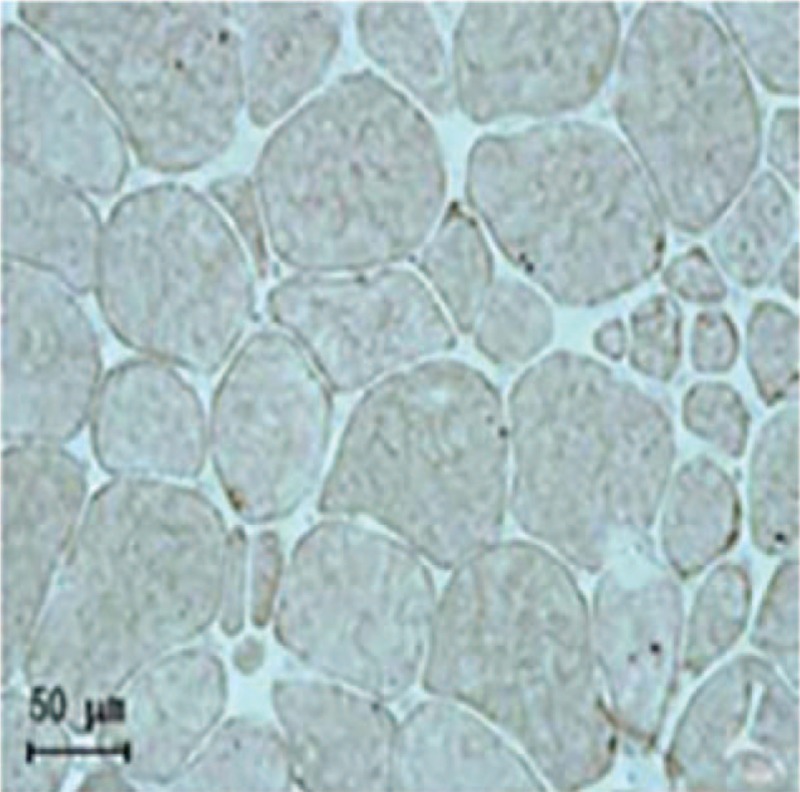
Dysferlin staining of muscle fiber in the left quadriceps of the patient presented no coloration in sarcolemma of muscular tissue.

Dysferlin was negative, indicating deficiency of this membrane protein; dystrophin-C,N,R was positive, indicating normal existence of this membrane protein; CD4 (±), CD8 (–), CD68 (±), MHC-1 (–). Pathological diagnosis: typical LGMD2B (Figs. 1 and 2).

**Figure 3:**
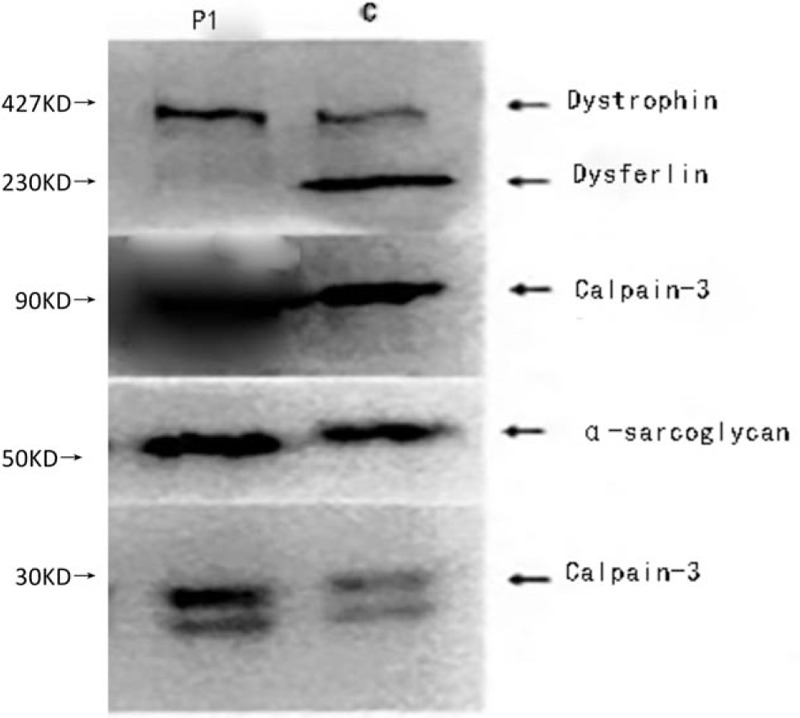
Western-blot analysis on the left quadriceps femoris of the patient indicated no expression of dysferlin protein band and normal expression of dystrophin, calpain-3 (exon), α-sarcoglycan, calpain-3 protein bands, P1: patient, C: control group.

**Figure 4:**
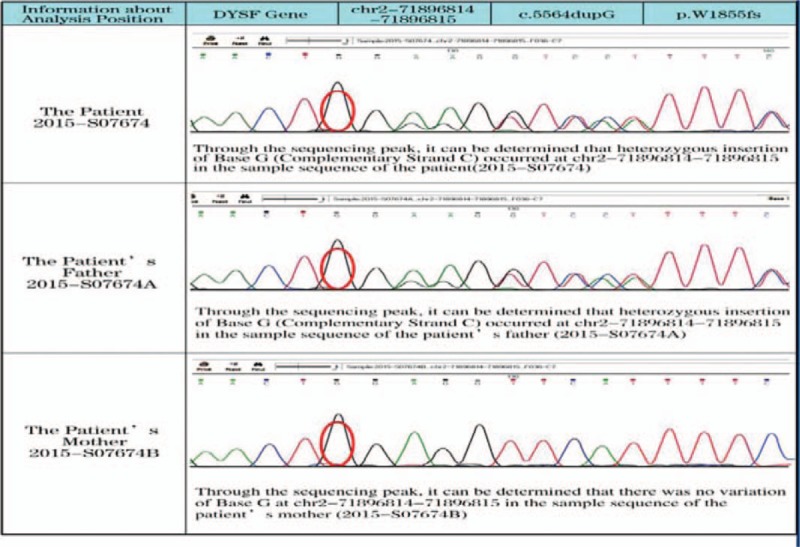
Pedigree analysis of the patient: For the patient, insertion of Base G occurred at chr2-71896814-71896815. For the patient's father, insertion of Base G also occurred at chr2-71896814-71896815. For the patient's mother, no variation of Base G at chr2-71896814-71896815 was detected.

**Figure 5:**
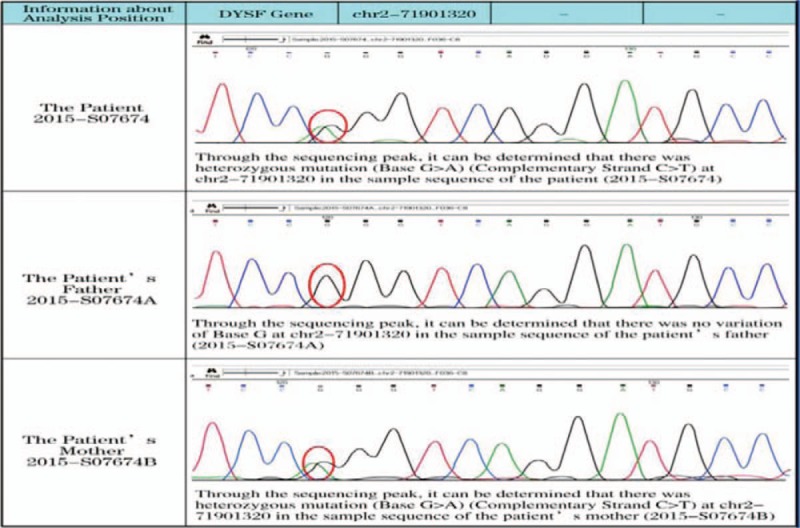
Pedigree analysis of the patient: For the patient, there was heterozygous mutation (Base G>A) at chr2-71901320. For the patient's mother, heterozygous mutation (Base G>A) at chr2-71901320 was detected. For the patient's father, no variation of Base G at chr2-71901320 was detected.

*WB analysis of the left quadriceps femoris*: no expression of dysferlin band and normal expression of dystrophin, calpain-3 (exon), α-sarcoglycan, and calpain-3 protein bands (Fig. 3). Figures 4 and 5 show the results of relevant gene testing of LGMD.

Multiple muscle enzyme examinations of the patient in other hospitals prompted multiple enzymatic changes. The highest level of creatine kinase (CK) reached 8448.2I U/L (26–140I U/L). EMG examination showed for many times myogenic changes, which prompt her diagnosis with PM, of which she was repeatedly treated with hormone and immunosuppressive agent. However, her symptoms had not been improved and gradually aggravated, so she came to our hospital. After relevant examinations, such as genetic testing, immunohistochemical examination, and WB analysis, she was diagnosed with LGMD2B, instead of PM. After symptomatic treatments such as coenzyme Q10, the patient's symptoms were obviously relieved, and the CK level decreased.

## 3 Discussion

This patient, a young woman, showed insidious onset of the condition; the main symptom was progressive aggravation of proximal muscle weakness of both lower limbs. Results of auxiliary examinations indicated obvious increased level of CK and amyotrophy of both lower limbs, and EMG indicated muscle-derived injury. Prior to the muscle biopsy and immunohistochemical exanimation, she was diagnosed with PM and treated with glucocorticoids and immunosuppressive therapy at various hospitals. After the treatment, there was no obvious improvement in the patient's clinical symptoms and CK level, and her symptoms worsened. After admission to our hospital, she underwent immunohistochemical examinations and WB analysis, which indicated deficiency of dysferlin and negative result for monoclonal antibody of MHC-1 and CD8 in sarcolemma, accompanied by point mutation and frame-shift mutation of *DYSF* gene. Based on the above results, the patient was diagnosed with LGMD2B.

PM is a CD8+T-mediated autoimmune disease with obvious symptoms affecting the proximal muscles of the four limbs. A common symptom is symmetrical weakness of the shoulder girdle muscles and anterior cervical extensor; both, the upper and lower limbs may be affected. With disease progression, distal muscle weakness and muscle atrophy may present. Facial muscles are rarely affected. In addition to myopathy, PM may also have other clinical manifestations, such as periorbital skin rash and circum-finger lesion. Muscle biopsy indicates degeneration and necrosis of muscle fiber, phagocytosis and cell regeneration, degeneration of basophilic cell, nuclear membrane enlargement, obvious nucleus, various sizes of fiber, and inflammatory exudation. In addition, the above manifestations may be accompanied by increased CK level and muscle-derived injury.^[3]^

LGMD2B is a common clinical phenotype in dysferlinopathy, for which the most obvious feature of histopathological examination is the obvious necrosis of affected muscles due to deficiency of dysferlin. At the early stage, the disease may simply present as splitting of muscle fibers. With disease progression, degeneration and necrosis of muscle fiber in the affected muscle group can be observed, accompanied by inflammatory cell infiltration.^[4,5]^ EMG indicates muscle-derived injury, while histopathological examination indicates deficiency of dysferlin.

Misdiagnosis of LGMD2B as PM is common. These two diseases have some common symptoms, including asymmetric weakness of limbs, increased CK level, and electromyographic changes indicating muscle-derived injury. In addition, results of histochemical pathological examination on skeletal muscle all present various degrees of myofibrosis, muscle fiber necrosis, and inflammatory cell infiltration. The main method for distinguishing these two diseases is analysis on expression of dysferlin in sarcolemma by IHC or WB analysis. For patients with LGMD2B, results of IHC or WB analysis indicate deficiency of dysferlin in the affected muscle fiber and negative result or low expression of MHC-I, but results of such analysis for PM patients are the opposite.^[6]^ Both IHC and WB analysis can be used to determine expression of dysferlin.^[1]^ Dysferlin is expressed in membrane and cytoplast of multiple types of cells, so WB analysis is more sensitive than IHC. At the early stage of such diseases, WB analysis can be used to determine expression of dysferlin. Utilization of IHC and WB analysis can increase sensitivity of diagnosis of LGMD2B and thus plays a significant guiding role in early stage treatment.

Through this case we found that LGMD2B and PM are very similar in clinical phenotype and auxiliary examinations; hence, it is very difficult to distinguish one from the other. However, therapeutic schemes for these conditions are totally different. PM is an immunological disease and responds well to hormone and immunosuppressive agent; thus, use of hormone or immunosuppressive agent is recommended at the early stage to control disease progression.^[7]^ However, in patients with LGMD2B, emphasis is placed on symptomatic treatment and appropriate exercises at the early stage, which can slow down disease progression and enhance maximum improvement of motor functions. In addition, some studies found that patients with LGMD2B may suffer from muscle weakness after being treated with hormones, and it was very difficult to regain muscle strength after stopping the hormone treatment, which indicated that use of glucocorticoids may aggravate the condition of patients with LGMD2B.^[8]^ Therefore, whether a definite diagnosis of LGMD2B can be made at the early stage is closely related to the prognosis of patients. Through IHC and WB analysis of tissues in affected muscles, diagnosis of LGMD2B can be effectively improved. For patients with suspected symptoms of PM who meet diagnostic criteria of PM, regular immunohistochemical examination on muscle is recommended. When result of IHC examination indicates partial deficiency of dysferlin, WB analysis should be used to detect dysferlin. If necessary, LGMD2B gene testing may be conducted for differential diagnosis between this disease and LGMD2B.

Meanwhile, this patient had *DYSF* gene mutation, and heterozygous mutation of *DYSF* gene (c.5621G>A) appeared at chr2–71901320, which was related to LGMD2B according to relevant reports. However, whether heterozygous mutation at this site is related to other neuromuscular diseases, such as PM, should be demonstrated and proved in relevant clinical tests. Meanwhile, Base G insertion was detected at chr2-71896814-71896815 in this patient. There is no relevant report about frame-shift mutation at this sit; hence, so further studies are needed to verify whether such mutation is related to occurrence of LGMD2B. It is difficult diagnosed LGMD2B simply through gene testing, but combination of gene testing, IHC and WB analysis is conducive.

At present, there is no special medicine for treatment of LGMD2B. Symptomatic treatment and limb motion treatment serve as the major therapies for LGMD2B. In in vitro experiments, gene editing technology was used for the treatment of multiple types of neuromuscular disease models including LGMD2B, and encouraging clinical effects were observed. However, such clinical effects in patients with LGMD2B should be verified by a large-scale clinical study.^[9,10]^ It is believed that promotion of immunohistochemical techniques and development of gene editing technology will play a revolutionary role in diagnosis and treatment of LGMD2B.

In this case, IHC and WB analysis were sufficient for diagnosis, but no WB analysis was conducted on other sarcolemma surface proteins such as dystrophin (N-end), dystrophin (C-end), and calpain3 60KD, so deficiency of such membrane surface proteins cannot be determined. At present, fewer clinical cases are diagnosed by combination of immunohistochemistry and WB analysis. In the future, not only should we use IHC and WB analysis in diagnosis of more patients with weakness in limbs, but also we will increase the number of types of sarcolemma surface proteins in immunohistochemistry and WB analysis. In this way, early diagnosis of LGMD2B will be further improved, and occurrences of misdiagnosis and missed diagnosis of this disease will be reduced.

## Author contributions

**Supervision:** Jiajun Chen, Jia Li.

**Writing – original draft:** chuan xu.

**Writing – review & editing:** Yingyu Zhang.
